# An automatic system for kinetic clinical analyses

**DOI:** 10.1155/S1463924679000250

**Published:** 1979

**Authors:** Clifford Riley, Colin F. Darby, David E. Tutt, Bernard F. Rocks

**Affiliations:** 1 Royal Sussex County Hospital Brighton UK; 2 Bioengineering Department University of Liverpool Liverpool L69 3BX UK; 3 University of Sussex Falmer Brighton UK; 4 Royal Sussex County Hospital Brighton UK


deleted, the remainder packed down, and new data entered at 4 sec intervals. This process of checking the $\Delta A$ between 'Time Zero' and the tenth data point, and then doubling the time base, can provide reading intervals of 2, 4, 8, 16, 32 and 64 sec on any cuvette. Calculation of the $\Delta A$ value and checking that it exceeds the predetermined minimum occurs when a signal is being acquired on the following cuvette. The advantage of this algorithm is that although the time base for individual reactions has a dynamic range of 64.2 within the overall monitoring period of between 18 sec and 34 min, only 10 data points are stored for any given cuvette.

The calculation routines for regression slope and standard error of reaction for the cuvette that is leaving the reading sector, are time shared with data acquisition prior to that cuvette being cleaned ready to receive a new sample at the sample transfer position. When the scanning beam leaves the reading sector, other computer controlled tasks begin. Printing of results is time shared with the foregoing mathematics package, and with a throughput of a fresh sample every 24 sec, the total time available for calculation and printing is 12 x 0.5 = 6 seconds, corresponding to 12 revolutions of the scanning beam and the time the beam spends in the non reading sector. A further process control task is that of counting the rotations of the scanning beam. This provides the necessary command pulse required every 12th revolution, to index the entire rotor forward on completing one cycle ready to receive the next sample.

### Discussion

The basic principle embodied in this experimental prototype of a scanning multiplexed absorptiometer working within a continuous feed preparative unit, allows for both high sample throughput and a wide range of reaction times. The system as described, with the absorptiometer functioning in a multiplexed scanning mode, can monitor reactions over the range of 18 sec-34 mins, but can be extended — at the expense of sample throughput — to monitor reactions over even longer periods (e.g. 45 min monitoring, at 100 tests per hour). Shorter observation times for fast reactions can be realised by mechanically locking the absorptiometer beam to the cuvette under investigation; this enables a reaction to be monitored from 'Time Zero' to 23 sec. Using the analogue processing techniques described, the data rate is limited to approximately 100 datum points per sec but the algorithm and mathematics package function in the same way as for slower reactions.

The principles of the system will be incorporated in a new instrument under development by Coulter Scientific Inc., Hialeah, Florida, USA. Preliminary results [8] obtained on the prototype were presented at the Tenth International Congress of Clinical Chemistry, Mexico City 1978. It is hoped that a system similiar to that described will become commercially available in the near future, providing the analyst with a high capacity analyser able to monitor reactions over periods longer than those systems currently in use.

ACKNOWLEDGEMENTS

The authors wish to thank Dr. S. S. Brown, Dr. F. L. Mitchell and Mr. S. N. Pocock for their encouragement and advice and would also like to acknowledge the significant programming contribution of Mr. G. Hardcastle.

REFERENCES

[1] Instrumentation Guidelines Study Group. American Association of Clinical Chemists 1977, 23, 2160.

[2] Greinke, R. A., Mark Jr., H. B., *Analytical Chemistry* 1978, 50, 70R Fundamental Reviews.

[3] Fishmann, M. M., *Analytical Chemistry* 1978, 50, 261R Fundamental Reviews.

[4] Tarbit, I., *Laboratory Equipment Digest* 1978, May, p.75.

[5] Avery, J., Gregory IV, R. P., Renow, B. W., Woodruff, T., Malmstadt, H. V., *Clinical Chemistry* 1974, 20, 942.

[6] Anderson, N. G., *American Journal of Clinical Pathology* 1970, 53, 778.

[7] Pardue, H., *Clinical Chemistry* 1977, 23, 2189.

[8] Horne, T., *Abstracts from X International Congress of Clinical Chemistry,* Mexico 1978, 57, Abstr. No. 4.

### An automatic system for kinetic clinical analyses

**Clifford Riley**
*Royal Sussex County Hospital, Brighton, U.K.*
**Colin F. Darby**
*Bioengineering Department, University of Liverpool, Liverpool L69 3BX, U.K.*
**David E. Tutt**
*University of Sussex, Falmer, Brighton, U.K.*
**Bernard F. Rocks**
*Royal Sussex County Hospital, Brighton, U.K.*

THERE is a considerable need for an analytical system so designed that the relatively simple tests needed urgently in hospital may be carried out quickly at short notice. The following describes a possible solution to this problem.

Such a system should be capable of performing tests extremely rapidly with a precision at least as good as that found with existing conventional machines. The 'steady state' methods normally employed in analytical machines are for the most part too time consuming to suit an emergency system; the need is to make measurements in a matter of thirty seconds or so, a requirement which can be met by using reaction kinetics. The merits of kinetic methods have been reviewed by Pardue [1], Malmstadt et al [2] and Moss [3] and the measurement in blood plasma of a number of substances, other than enzymes, using such methods have been described; creatinine, (Cook [4] and Fabiny et al [5]), cholesterol, (Hewitt et al [6]), glucose, (Kadish [7]), urea, (Hallett et al [8]), thyroxin-eiodine, (Areq et al [9]) and amylase, (Shipe et al [10]). There is little doubt that kinetic methods can be at least as precise as traditional methods and indeed possess intrinsic advantages such as the reduced need to run blanks and short analysis time.

It is essential that such equipment can be left on standby for prolonged periods. Fortunately, modern opto-electronic systems are very stable and data can be stored indefinitely in digital registers.

An additional important requirement for such equipment is that it should be easy to use, so that if necessary, it will give precise

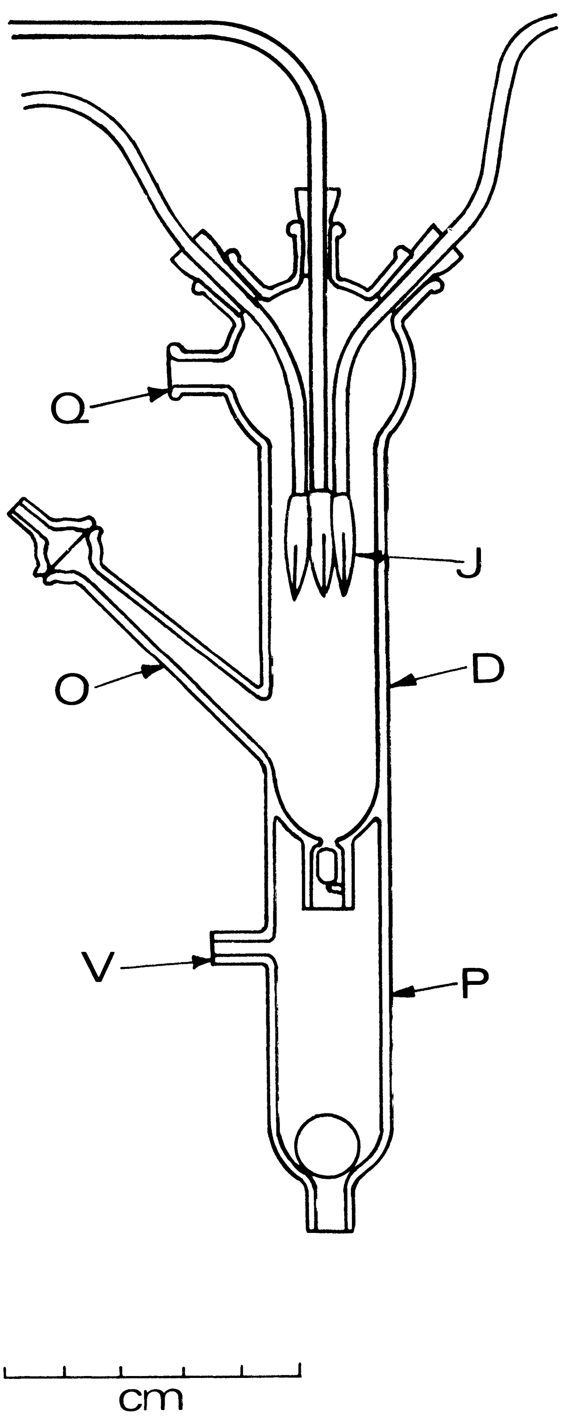

*Figure 1. Reaction vessel.*

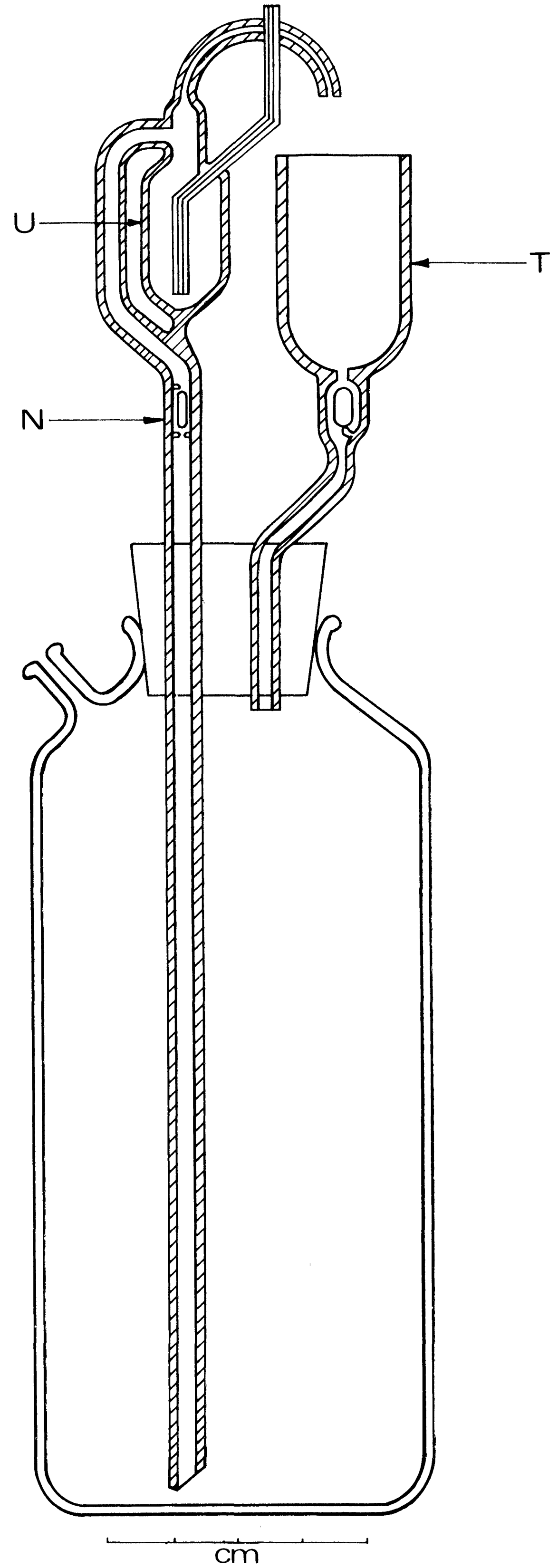

*Figure 2. Reagent dispenser.*

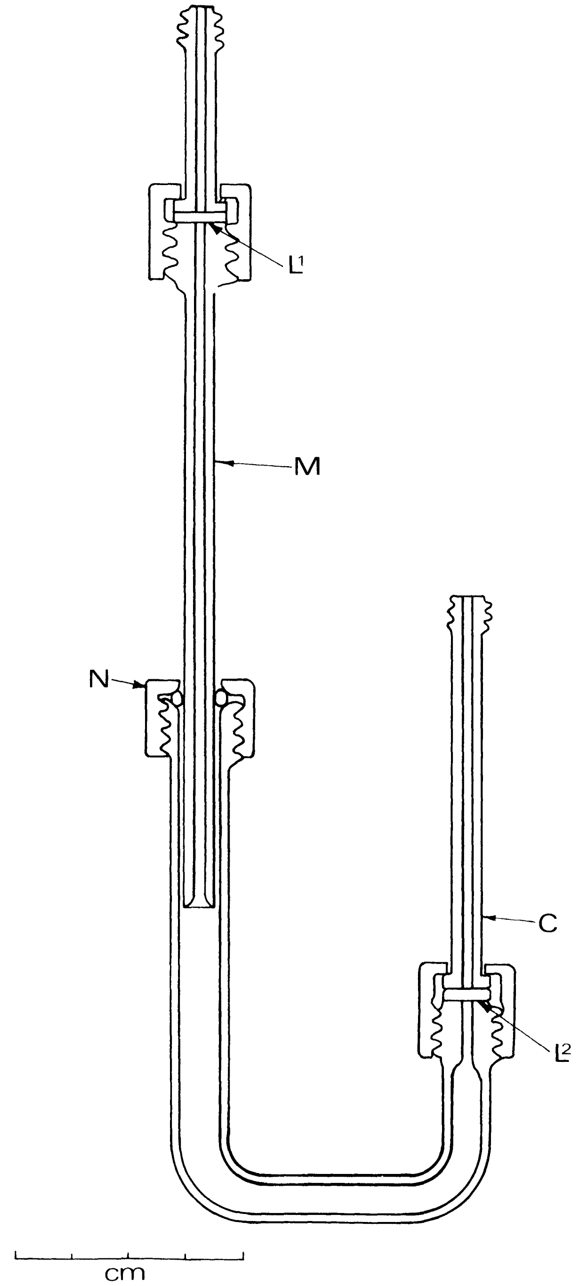

*Figure 3. Sample measuring unit.*

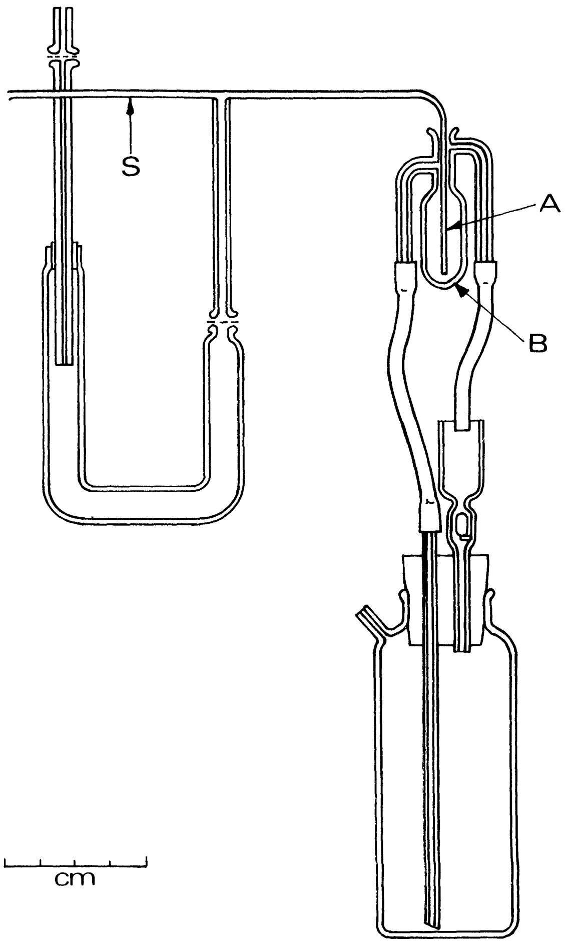

*Figure 4. Sample measuring system.*

results in the hands of untrained personnel. To achieve this the operator should have little more to do than offer a sample to the machine and press a button.

The problem therefore was to devise a system which met all these requirements. In addition it would have to be cheap to manufacture, since there are practical advantages in building on a modular basis with one module dedicated to each analysis.

### The fluid handling system

The model originally developed was in due course extensively modified but it will be described first as its working is easier to follow. In order to avoid expensive construction and to achieve great reliability, the use of moving parts such as syringes and pumps was avoided as far as possible and fluid handling achieved by using a combination of vacuum and compressed air controlled by a cam timer. This system was constructed of chemically resistant materials. The reagent bottles were of high density polyethylene, the reagent lines were of PTFE and all the special glassware was made of borosilicate glass.

The central unit is the reaction chamber into which, by applying vacuum, previously measured sample, diluent and reagents can be drawn. This is shown in Figure 1. D is the chamber in which the reaction takes place. Reagents and sample can be drawn in through the small bore tubes at the head of the chamber by applying vacuum to the side arm Q. The lower chamber P provides drainage to the reaction chamber; a preliminary to each reaction sequence is to pressurise this chamber through V in order to seat both ball valves. At the end of each cycle of operations the reaction chamber is opened to atmosphere and vacuum briefly applied to the lower chamber in order to unseat both ball valves. The lower ball is less dense than water so that it remains unseated until both chambers have drained. The outlets J are very simple 'duckbill' type non-return valves moulded in silicone rubber.

The side arm O of the reaction chamber provides vibrational stirring in the following way. It is closed with a rubber diaphragm which is connected by tubing to the head of the air compressor which supplies the whole system. The fluctuating air pressure causes the diaphragm to pulsate at pump speed and this causes oscillation of the column of liquid in the lower end of the side arm O.

Reagent dispensers to deliver a range of volumes were constructed as shown in Figure 2. Brief application of pressure to the reagent bottle fills the pipette-like vessel U and surplus reagent overflows through the constricted neck into the funnel T. When the pressure in the reagent bottle drops to atmospheric the non-return valve in the funnel T opens and allows surplus reagent to drain into the bottle. A fine capillary which extends to the bottom of chamber U allows the measured volume of reagent to be drawn over into the reaction chamber.

The measurement of the sample was necessarily more complex. To meet the requirements of the system the measured sample needed to be aspirated, and to avoid carryover is followed by a measured volume of diluent.

The device for aspirating the measured volume of sample is shown in Figure 3. This is a U-tube with an adjustable capillary limb M. $L_1$ and $L_2$ are sintered glass of fine porosity. The capillary limb M has a bore of 1 mm and can be adjusted for height through the gland N. The U-tube is filled with mercury up to the lower end of the capillary. When vacuum is applied to the upper end of the capillary arm, the mercury is drawn up until it is stopped by the sintered disc $L_1$. When the vacuum is broken the mercury flows back until it is arrested by the sintered disc $L_2$. During this part of the cycle of operations, distilled water in limb C is drawn in and subsequently ejected through $L_2$ in a volume determined by the length of capillary M. This unit thus functions in much the same fashion as the sample syringe in a two syringe dilutor but without the need for any moving parts.

The complete sampling and diluting assembly is shown in Figure 4. The PTFE probe A normally rests in a small vessel B which is filled with distilled water to constant volume in the same manner as the dispenser. The vessel is mounted on a lever so that it can be moved away from the probe and a sample may be offered to it. The sample line S terminates in a non-return valve inside the reaction chamber (see Figure 5). In use, the diluent vessel is withdrawn, a sample of plasma or standard offered to the probe and vacuum applied to the capillary end of the U-tube. A measured aliquot of sample is thus drawn into the sample line. The diluent vessel is replaced in position so that when vacuum is applied to the reaction vessel the sample, followed by a measured volume of diluent, is drawn into it.

The complete assembly is shown in Figure 5. In operation the diluent reservoir is moved away from the probe A, a sample is offered up to the probe and the system set in motion by pressing the start button. Pressure is applied briefly to the reagent and diluent bottles and continually to the lower chamber of the reaction vessel. At the same time vacuum is applied to the capillary limb of the U-tube. Thus diluent and reagent dispensers are filled, the valves of the reaction vessel are closed and a measured aliquot of plasma or serum is drawn in the probe. The diluent reservoir is replaced and this initiates the rest of the cycle. Vacuum is applied to the reaction chamber drawing in reagents and sample, followed by diluent. The vacuum on the U-tube is broken during this stage, allowing the mercury to fall back to its original level and the measured aliquot of distilled water to follow the rest of the diluent.

The stirred reaction mixture is observed for an appropriate period of time — usually 30 to 45 seconds — and the system is then drained and rinsed as follows. The vacuum on the reaction chamber is broken; then vacuum is drawn briefly on the lower chamber allowing the system to drain. Then the lower chamber is pressurised to seat the valves, vacuum is drawn on the reaction chamber and simultaneously the bottle of diluent is pressurised for several seconds. As a result, several millilitres of diluent (distilled water containing non-ionic detergent) is drawn into the reaction chamber.

The system is drained again and the wash cycle repeated. It was found experimentally that two wash cycles were sufficient to reduce carryover to undetectable levels.

Although the system worked well, after experience it was found that some modifications improved reliability. For example the ball valve at the lower end of the reaction chamber did not always seat properly; so, it was finally replaced by a thick disc of silicone rubber, provided with a suitable seating which now always gives a gas-tight seal. Foaming sometimes occurred in the lower chamber and when vacuum was drawn, foam found its way into the control system. The lower valve was therefore arranged so that it was lifted by vacuum, but after lifting a short distance it encountered a second seating and closed off the vacuum. The diaphragm of the stirring system sometimes became contamin-

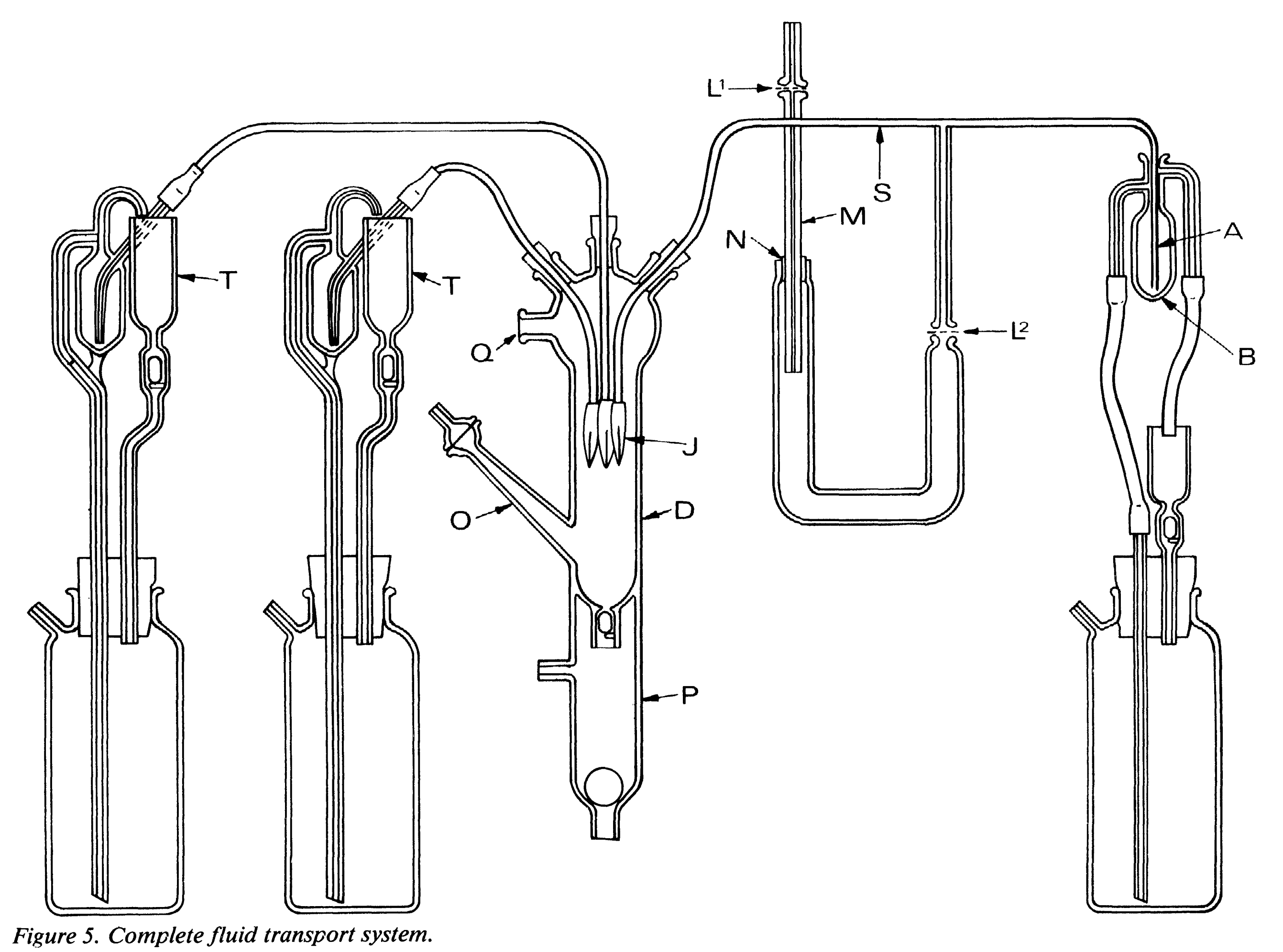

*Figure 5. Complete fluid transport system.*

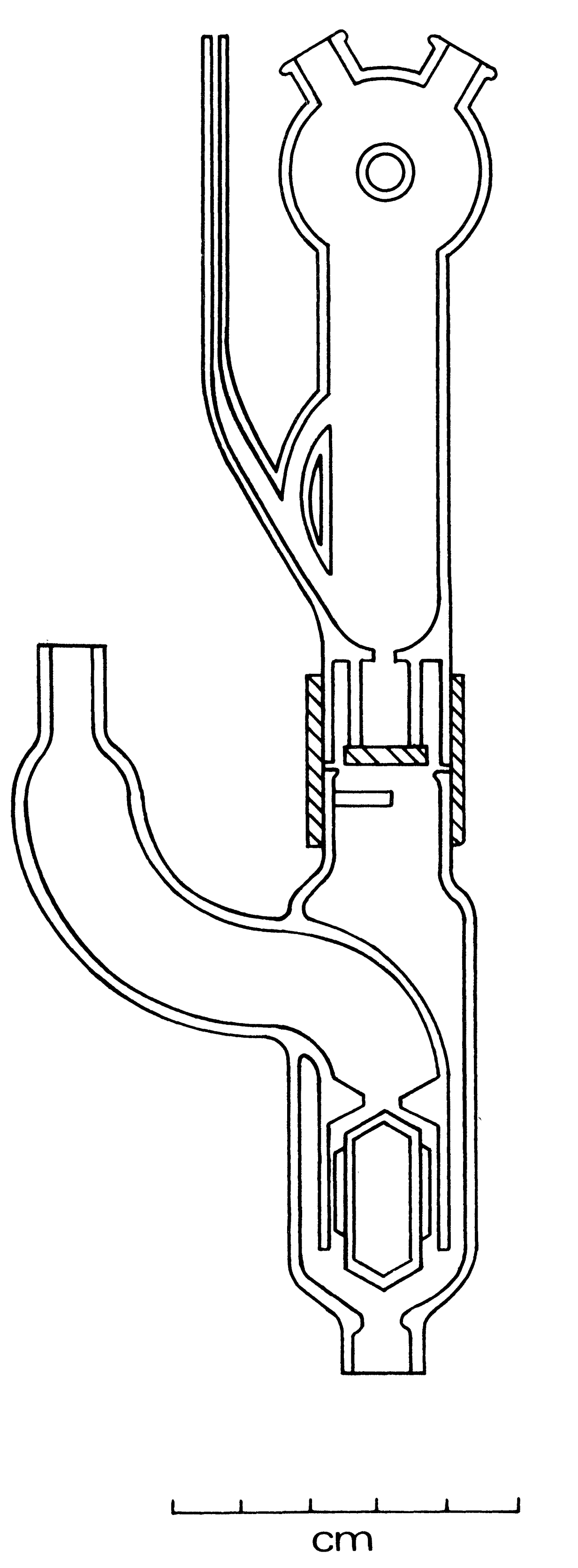

*Figure 6. Modified reaction vessel.*

ated with the reaction mixture so this stirring system was removed and replaced by an air leak tube. The modified reaction vessel is shown in Figure 6.

It was felt that there might be a need to delay the addition of one or more reagents. The reagent dispensers were therefore enclosed in glass envelopes on which vacuum could be drawn simultaneously with that on the reaction chamber. The reagent in the dispenser thus remained in situ until the vacuum to its envelope was broken. The provision of these envelopes was also found to prevent reagents from crystallising out in the unit when the system was out of use for prolonged periods.

The mercury U-tube proved to be a maintenance problem because the sintered discs became blocked after two or three weeks, and it was virtually impossible to change them without spilling mercury. This device was therefore replaced by a diaphragm operated displacement sampler as shown in Figure 7. The diaphragm and its housing is a commercially made pneumatic valve actuator (1). The block in which the displacement rod operates is machined from perspex, the rod is of ground stainless steel and the seal is provided by a silicone rubber 'O' ring.

Finally, the system of providing diluent was modified. It was unsatisfactory that surplus diluent, contaminated with plasma from the outside of the probe, was returned to the reservoir. However, running surplus diluent to waste meant a much larger reservoir and as this emptied, it required prohibitively large volumes of compressed air to operate it. This difficulty was overcome by the provision of a constant level 'chicken feed' device as shown in Figure 8.

The complete modified fluid transport system is shown diagramatically in Figure 9. In this figure it will be noted that the 'duckbill' valves have been dispensed with; during operation of the sampling device the latter is isolated by a pair of Tufnol blocks (T) which pinch off a segment of silicone rubber tubing when the diluent reservoir is lowered.

The reproducibility of the fluid measuring devices was evaluated with water, by repeatedly sampling or dispensing and weighing the transferred liquid. The nominal 1.0 ml dispenser gave a mean of 1.004 ml and a coefficient of variation of 0.63 whilst the nominal 4.0 ml dispenser gave a mean of 3.88 ml and a coefficient of variation of 0.14. The diluent dispenser nominally 1.0 ml gave a mean of 0.90 ml and a coefficient of variation 0.56 The mercury sampler, sampling nominally 100 µl gave a mean of 92.90 µl and a coefficient of variation of 0.45%. The mechanical displacement sampler, sampling nominally 75 µl gave a mean of 72.25 µl and a coefficient of variation of 0.43%.

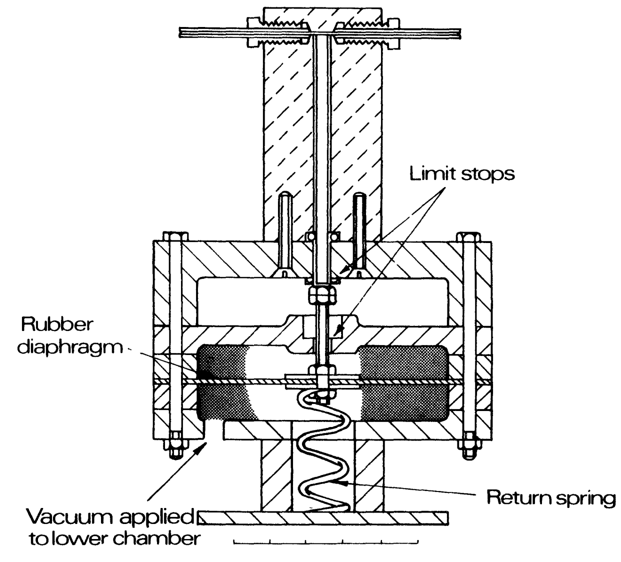

*Figure 7. Diaphragm operated displacement sampler.*

### The control unit

It was originally intended to construct a solid state control unit with electromagnetic valves as an interface with the pneumatic system. It was obvious however, that this would be costly and an inexpensive cam timer was substituted. This was equipped both with microswitches and microvalves (2) to control supplies of compressed air to various units. Since the microvalves could not be used to control vacuum, where control of vacuum was needed, microvalves were used to control the air supplied to 'Powerlog' pneumatically operated spool valves (3). Vacuum (approx. 100 mm Hg) and compressed air (10 psi) were provided by a two stage diaphragm compressor (4). The two stages were disconnected, the first used to provide compressed air and the second to provide vacuum. The cycle time of the control unit was originally two minutes but experience showed that this could be reduced to ninety seconds without loss of precision.

### The photometric unit

This was specially designed to measure the low light levels obtained in chemiluminescent reactions and also to provide facilities for fluorescence and absorbance studies. As a photodetector a 10 mm PIN diode was used instead of a photomultiplier. This device is linear over many decades of light intensity, is cheaper to install than a photomultiplier and most important, works at low voltages. It was however, necessary to mechanically chop the light in order to obtain sufficient amplification for signals from chemiluminescence reactions. Multilayer interference filters were used for work in fluorescence and colorimetry The light source was a quartz iodine projector lamp which was effective for the measurement of NADH since it gave ample emission at 340 nm.

### Data handling system

Two digital registers were provided, one of which accepts a standard value and retains it as long as necessary and the other

*(1) Schrader. Industrial Fluid Power Products, Bridgetown, Staffs.*
*(2) Crouzet Ltd. Brentford, Middlesex.*
*(3) Desoutter Lang Ltd. Halesfield, Telford, Shropshire.*
*(4) Charles Austen Pumps Ltd. Byfleet, Surrey.*

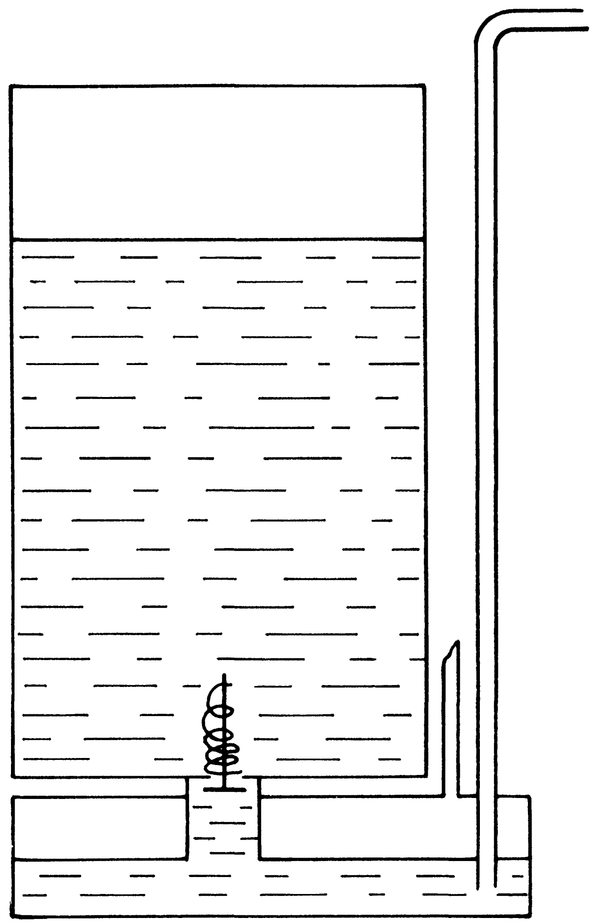

*Figure 8. Modified diluent container.*

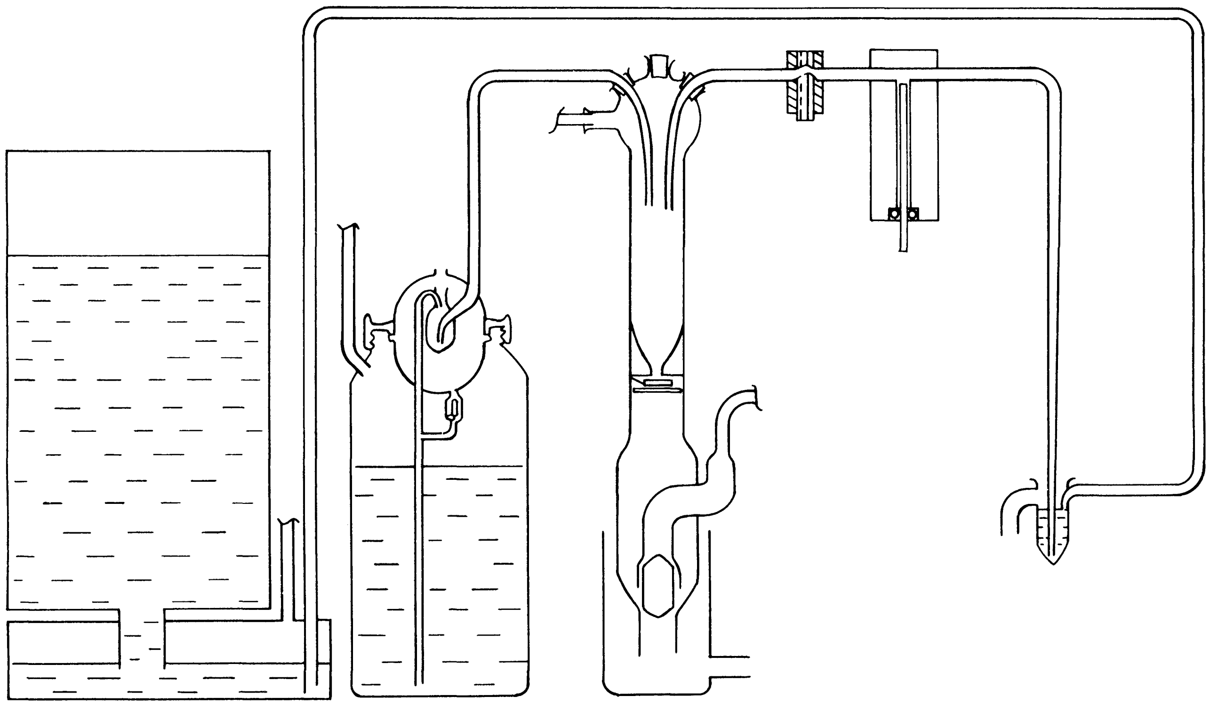

*Figure 9. Modified fluid transport system.*

accepts signals from the unknown sample. There are facilities for inserting the standard value and the system automatically carries out the calculation $\frac{\text{test reading}}{\text{std. reading}}$ x standard value. There are also logarithmic facilities available and other linearising facilities have been considered but have not so far been needed. Work is in hand to replace this system and the control unit by a microprocessor.

The entire fluid handling system together with the photometric unit was housed in a steel cabinet well insulated with polystyrene foam and thermostatted at 35° ± 0.2°. The system has been tested by estimating serum glucose with glucose oxidase in conjunction with a chemiluminescent reaction and total serum protein by a biuret method, (Weischelbaum [11]) but operating at 30°.

Chemiluminescence is a phenomenon that deserves more exploitation as a means of making kinetic observations, since the light level obtained is directly proportional to the rate of the reaction. In the method used, liberated hydrogen peroxide was allowed to react with an acridinium salt and the light emitted measured at 425 nm. This method was developed by one of us (DET) and will be fully described elsewhere.

The reproducibility of the glucose method was excellent; 20 replicate aqueous samples of nominally 11.1 mmol/l gave a mean of 11.09 mmol/l and a coefficient of variation of 0.62%. This method is linear to within 1% up to 33.3 mmol/.

46 samples of plasma preserved with fluoride were measured, both by the chemiluminescent and hexokinase methods and gave a correlation coefficient of 0.996. The only disadvantage of the method is the relatively large volume (60 µl) of plasma needed for each test. For measurement of total protein, the machine was used in the absorbance mode with the arithmetic unit equipped with an analogue logarithmic converter. Assays of 20 replicates of bovine albumin 80 g/l gave a mean of 80.33 and a coefficient of variation of 0.45%. It is interesting to note that the error in this analysis is very little more than that found in the sampler itself so that other measuring units make little contribution to the total error. The machine showed no departure from linearity up to 120 g/l. 24 plasma samples with total protein ranging from 42 to 88 g/l were assayed both by the emergency machine and the Vickers M300 and a coefficient of correlation of 0.980 was obtained.

A number of other analytical methods have been tried under simulated conditions but have not yet been used on the machine. These include a fluorimetric rate method for glucose; a fluorimetric rate method for alkaline phosphatase and a urease urea method using conductivity to measure the rate of liberation of ammonia.

ACKNOWLEDGEMENTS
The authors are grateful to Mr. A. Hoare of A. W. Dixon & Co., Avenue Road, Beckenham, Kent, who made all the glassware gratis. Funds for this development were provided by the Wellcome Foundation.

REFERENCES

[1] Pardue, H. L. in *Recent Advances in Analytical Biochemistry* Vol 7, 1969 Interscience.
[2] Malmstadt, H. V., Delaney, C. J., Cordes, E. A. *C.R.C. Critical Reviews in Analytical Chemistry* 1972, **2**, 559.
[3] Moss, D. W. *Clinical Chemistry* 1972, **8**, 1149.
[4] Cook, J. G. H. *Clinica Chimica Acta* 1971, **32**, 485.
[5] Fabiny, D. L., Ertingshausen, G. *Clinical Chemistry* 1971, **17**, 696.
[6] Hewitt, T. E., Pardue, H. L. *Clinical Chemistry* 1973, **19**, 1128.
[7] Kadish, A. H., Little, R. L., Sternberg, J. C. *Clinical Chemistry* 1968, **14**, 116.
[8] Hallett, C. J., Cook, J. G. H. *Clinica Chimica Acta* 1971, **35**, 33.
[9] Arcq, J. C., Arcq, A. *Clinica Chimica Acta* 1973, **48**, 287.
[10] Shipe, J. R., Savory, J. *Clinical Chemistry* 1972, **18**, 1323.
[11] Weichselbaum, T. E. *American Journal of Clinical Pathology*, Technical Section 1946, **10**, 40.

### The design of a microprocessor system for automatic signal averaging and percentage purity calculations coupled to a nuclear magnetic resonance spectrometer

**F. Morley, I. K. O'Neill*, M. A. Pringuer**, and P. B. Stockwell**
*Laboratory of the Government Chemist, Cornwall House, Stamford Street, London SE1 9NQ.*

NUCLEAR magnetic resonance (NMR) spectroscopy is inherently a quantitative technique and the area of a group of peaks is therefore directly related to the concentration. The practical problem is the reliable integration of the area under the peak. Digital integrators are readily available for gas and liquid chromatographic applications but as yet none has been specifically designed for NMR spectroscopy. In contrast to gas chromatography, where gaussian shaped peaks are the ideal, NMR peaks have a considerably faster slew rate and require different integration criteria to measure the correct area, whilst fine structure provides useful identification data it is the total area within the multiplet that is required for quantitation. An integrator specifically designed to measure the area under NMR peaks with the fine structure of the peak retained has been designed by Bunting[1, 2]. This paper describes the application of a microprocessor system to control both the spectrometer and the integrator to measure the areas under selected peaks, the use of multiscan averaging to improve signal to noise characteristics and to automatically calculate the percentage purity of a sample. A single scan of the NMR spectrum exhibits random noise characteristics which can be improved by repetitive scans to the extent of $N^{1/2}$ where N = number of scans.

Methods have been developed in this laboratory to analyse pharmaceuticals quantitatively by NMR and an evaluation of the microprocessor controlled system described in this paper has been published by Pringuer et al [3]. A JEOL 60 $MH_z$ NMR

* *Presently on secondment to RTZ Services Ltd., York House, Bond Street, Bristol BS1 3PE.*
** *Present address, Life Science Research, Stock, Essex.*

